# AI adoption among adolescents in education: extending the UTAUT2 with psychological and contextual factors

**DOI:** 10.3389/frai.2025.1614993

**Published:** 2025-09-08

**Authors:** Luca Ballestra Caffaratti, Claudio Longobardi, Laura Badenes-Ribera, Davide Marengo

**Affiliations:** ^1^Department of Methodology of Behavioral Sciences, Faculty of Psychology and Speech Therapy, Universitat de València, València, Spain; ^2^Department of Psychology, University of Turin, Turin, Italy

**Keywords:** artificial intelligence in education, UTAUT2 framework, adolescent technology use, problematic internet use, behavioural intention

## Abstract

**Introduction:**

This correlational study investigates the psychological and contextual factors associated with the adoption of artificial intelligence (AI) technologies among Italian high school students. Building on the Unified Theory of Acceptance and Use of Technology 2 (UTAUT2), the study extends the model by incorporating Problematic Internet Use (PIU) and Attitudes Toward AI (ATAI) to better account for habitual AI use and behavioural intentions.

**Method:**

A sample of 933 students (M_age_ = 16.20, *SD_age_* = 1.29, 54.98% female) completed a survey assessing key UTAUT2 dimensions, psychological traits, and usage patterns of AI tools in educational contexts. Confirmatory factor analysis (CFA) was used to evaluate the functioning of the adapted UTAUT2. Multiple regression was used to investigate factors predicting habit formation and behavioural intention related to AI use.

**Results:**

Confirmatory factor analysis supported the structural validity of the adapted UTAUT2 model. Multiple regression analyses revealed that Performance Expectancy, Social Influence, Hedonic Motivation, and Schoolwork-related AI use were significant predictors of both habit and behavioural intention. PIU showed a robust association with habitual use, suggesting a spillover effect from compulsive Internet behavior to AI engagement. ATAI was associated only with behavioural intention, indicating its role in initial adoption rather than sustained use. Demographic and contextual factors (e.g., school type, citizenship) showed additional effects.

**Discussion:**

These findings contribute to a more comprehensive understanding of adolescent AI engagement by highlighting the role of compulsive tendencies and motivational beliefs. The study underscores the importance of designing inclusive, age-appropriate interventions to promote balanced and informed AI use in educational settings.

## Introduction

Artificial Intelligence (AI) technologies have become increasingly pervasive, shaping how individuals interact with digital environments (Moravec et al., 2024). This is particularly true for adolescents, who are at the forefront of using these tools due to their widespread exposure to the digital world, but who may lack the maturity to manage the associated risks. From productivity tools to creative applications, AI systems offer novel capabilities that are transforming educational, professional, and personal domains. As these technologies gain widespread adoption, understanding the factors influencing their use and the resulting behavioural patterns is critical, particularly among adolescent high school students who represent future workforce entrants and early adopters of technological innovations (Ryzhko et al., 2023).

In education, AI promises to lay the foundation for a profound redefinition of teaching practices and learning processes, opening up both new opportunities and significant challenges. While the development of AI holds significant potential to improve the quality of education, its impact is not without uncertainties and risks, necessitating a critical reflection on the responsible and balanced use of AI in education (AIED). An important aspect of this shift is to understand not only how AI works, but also how its use impacts students, with the aim of maximizing its benefits and mitigating potential negative effects, especially for younger users. In this context, high school students constitute a unique demographic for studying AI adoption. Adolescents are in a transitional phase characterized by identity formation and cognitive development (Erikson, 1968). At the same time, they are growing up in a digital environment (Prensky, 2001), which makes them early adopters and potentially vulnerable users of new technologies. In this phase, their technological engagement often intersects with educational tasks, social interactions and entertainment, making them an ideal population to explore AI usage patterns and attitudes (Ali et al., 2024). Existing models, such as the Unified Theory of Acceptance and Use of Technology (UTAUT2), provide a robust framework for examining the determinants of technology adoption, integrating constructs such as Performance Expectancy, Effort Expectancy, and Hedonic Motivation (Tamilmani et al., 2021). However, the application of UTAUT2 to the context of AI usage among adolescents remains underexplored, as do the nuanced roles of demographic, contextual, and psychological factors in shaping AI-related behaviors as possible expansions of the model itself (Ali et al., 2024; Cabrera-Sanchez et al., 2021).

### Artificial intelligence in education

The connection between AI development and education has deep roots that go back to the early stages of AI research. Cognitive scientists such as Simon and Newell combined their studies of machine learning with a deep interest in understanding human learning processes. These scientists saw AI not only as a technical innovation, but also as a key tool to advance educational theories (Doroudi, 2023). Over time, the focus gradually shifted from the pursuit of “Strong AI,” an AI designed to replicate human cognitive abilities (Searle, 1990), to the development of more practical and targeted systems known as “Weak AI.” These systems are designed to emulate human outcomes in specific domains. The introduction of tools such as ChatGPT, the first Generative Artificial Intelligence (GAI) platform, which has been freely accessible since November 2022, marked a turning point. AI use among adolescents has increased rapidly, especially in the education sector, where this age group has emerged as one of the key user demographics (Mogavi et al., 2024). According to Klarin et al. (2024), ChatGPT is currently the most used AI tool among adolescents, primarily used to complete school assignments. Apart from being the first GAI model introduced to the market, its popularity can probably be attributed to its ease of use, versatility and effectiveness in text generation, key features of technologies based on large language models (LLMs).

As a result of the growing accessibility of AI systems and their technological advances, academic research on AIED has experienced significant growth, with a notable increase in publications (Xia et al., 2023; Klarin et al., 2024). This growth reflects not only the increasing adoption of these technologies, but also the growing recognition of their transformative potential in education (Roy and Swargiary, 2024).

In the school environment, AI has the potential to be used for tasks such as translating, writing texts, and completing assignments. It may introduce new opportunities for personalized learning, by providing adaptive and tailored experiences for each student. For example, AI could be used to adapt educational content to the specific needs of individual students, especially in critical areas such as science, technology, engineering, and mathematics (STEM) (Panjwani-Charania and Zhai, 2023). AI support could be particularly valuable for students who face challenges, such as students with disabilities, learning disabilities, or language disadvantages, as they can benefit from support that is tailored to their unique characteristics and needs (Reiss, 2021). These applications could promote the integration of students with different cultural and linguistic backgrounds in the classroom (Salas-Pilco et al., 2022). AI systems can also be used to provide individualized tutoring by responding to students’ questions and offering explanations on complex topics, adapting the level of detail and language to each student’s needs (Roy and Swargiary, 2024).

### Risks, challenges, and conflicting findings

Beyond the positive aspects, several researchers have expressed significant concerns about the potential risks of AI use, highlighting in particular issues related to students’ digital health, wellbeing, and cognitive development. Some authors warn that excessive reliance on AI tools could encourage superficial learning habits and hinder the development of fundamental skills, such as critical thinking (Mogavi et al., 2024). If students consistently rely on AI to complete tasks or find solutions, they might not fully develop their creativity and independent thinking skills, potentially compromising the achievement of meaningful goals, such as independent writing (Costa and Murphy, 2025). The systematic use of AI could therefore pose risks to the development of important cognitive skills, such as planning and problem solving (Klarin et al., 2024).

As this is a relatively new and rapidly developing field, research on the psychological and social impact of students’ use of AI provides ambivalent and often contradictory results. Some studies emphasize the risks associated with AI, particularly the negative impact on students’ health, digital wellbeing and socio-emotional development. Others, however, emphasize the potential of AIED and present it as a significant opportunity for learning and personal development.

Several researchers warn of the risks of technological dependency resulting from the overuse of AI in educational activities. Klarin et al. (2024) and Mogavi et al. (2024) warn that the reliance on AI for educational and academic tasks could lead students to become overly dependent on these tools. Similarly, León-Domínguez (2024) suggests that the unregulated use of AI could even lead to situations of technology addiction. On the other hand, studies such as that of Roy and Swargiary (2024) indicate that student motivation and engagement increase in AI-supported learning activities, suggesting that these tools could promote more active and participatory learning experiences.

The effects of AI on students’ empathy and socio-emotional skills also show conflicting perspectives. Lai et al. (2023) find that emotional awareness and the ability to manage emotions decrease in adolescents who frequently use AI, raising concerns about their emotional development. In contrast, Wang et al. (2024) suggest that AI, by offering students personalized and inclusive learning, could contribute to developing greater social sensitivity, laying the foundation for potential openness to different cultures and perspectives.

At the relational level, there are different views on the impact of AI. On the one hand, Neugnot-Cerioli and Muss Laurenty (2024) point out the risk that prolonged use of AI could hinder the development of meaningful relationships in the real world and possibly lead to social isolation. On the other hand, a study conducted in China by Xie et al. (2022) offers an opposing perspective: students who use AI show higher scores in social adaptability scores compared to peers who do not, suggesting that AIED may actually improve social integration and adaptability. Furthermore, Lai et al. (2023) note that the use of AI could reduce direct interactions between students and educators, thereby limiting opportunities for socio-emotional learning. However, Neugnot-Cerioli (2024) suggests that AI could provide an inclusive learning environment for students with communication barriers, such as students with autism spectrum disorders, by providing them with tools to overcome relational barriers, thereby supporting their participation and social development.

The development and use of AI is a transformative phenomenon that is rapidly expanding and involving an ever-increasing number of people. AIED presents adolescents with scenarios that are rich in potential and at the same time raise critical questions. It is important to carefully assess the psychological and educational implications of the use of AI among adolescents, especially given the delicate developmental stage they are in. On the one hand, AI appears to offer tools for personalized learning, potentially opening new avenues for more effective educational outcomes. It could also hold promises for overcoming language and cultural barriers, potentially promoting inclusion in diverse school environments. On the other hand, concerns have been raised about the potential risk of technological dependency and the possible long-term impact on the acquisition of important skills and overall wellbeing. While research on AIED is still at an early stage, several studies raise important questions about the psychological and social impact of intensive and prolonged use of AI.

Enthusiasm for new technological possibilities must therefore be tempered by critical reflection on potential risks. Policy makers and educational institutions are at the forefront of addressing these challenges. The Artificial Intelligence Act, recently adopted by the European Parliament (2023), emphasizes the strategic importance of AI in education. Similarly, UNESCO has published a framework for AI competences for students at promoting the inclusive, sustainable, and responsible use of these technologies (Miao and Cukurova, 2024). This student-focused framework emphasizes the need to train students not only in the technical use of AI, but also in the critical understanding of its social and environmental impacts. The guide is structured around two main dimensions: the development of fundamental competencies.

### Classical models of technology acceptance and adoption

In recent decades, technology acceptance models have served as a basic theoretical framework for explaining the reasons why people choose to adopt new technologies. An important starting point is the Theory of Reasoned Action (TRA) developed by Fishbein and Ajzen (1975). According to TRA, individuals act on the basis of behavioural intentions, which are primarily derived from two factors: attitude towards the behavior, i.e., how positively or negatively a person evaluates the performance of a certain action, and subjective norms, i.e., the perceived expectations of people who are considered important (such as friends, family members or teachers) with regard to this behavior. If a person believes that important people expect them to perform a certain behavior, this belief will influence their decision to perform it or not in practice.

Building on these concepts, Ajzen (1991) extended the TRA by formulating the Theory of Planned Behavior (TPB), introduces the notion of perceived behavioural control, which is the individual’s belief as to whether they have the means to actually perform an action using the technology. Based on these premises, Davis (1989) developed the Technology Acceptance Model (TAM). This model identifies two main factors for the use of technology: perceived usefulness, i.e., how much the individual believes the technology will improve their performance, and perceived ease of use, i.e., how easy the user believes the technology will be to use. According to the TAM, these factors explain why a person intends to use technology in everyday life or at work.

To overcome the limitation of separate models, Venkatesh et al. (2003) proposed the Unified Theory of Acceptance and Use of Technology (UTAUT), which summarizes and integrates the key constructs from TRA, TPB and TAM into a single framework combining individual elements with contextual factors to explain technology adoption decisions. UTAUT identifies several key constructs to measure technology acceptance: Performance Expectancy, i.e., the perceived usefulness of a technology in improving one’s performance (e.g., a student evaluates whether AI can help to learn better or faster); Effort Expectancy, which refers to the perceived ease of use of the device; Social Influence, which measures how much the opinion of significant others (friends, teachers, parents) influences the decision to use the technology; and Facilitating Conditions, which is the extent to which the individual believes they have the resources and technical support they need to use the device.

The further development of this model, known as UTAUT2 (Venkatesh et al., 2012), later included additional factors such as Hedonic Motivation, Habit and Price value to also interpret technology use in the context of daily use (Tamilmani et al., 2021). Hedonic Motivation refers to the pleasure and curiosity associated with using technology; Habit is understood as the routine and continuous use of technology; price value represents the balance between the perceived benefits and the costs borne by the user. Recent studies focused on university students (Soliman et al., 2024; Soliman et al., 2025) have also confirmed that UTAUT2 provides a robust structure for modelling the adoption of AI tools in educational contexts.

### Integration of PIU and ATAI into the UTAUT2 model

Recent studies show the importance of considering psychological variables to explain complex behaviors such as the repeated and sometimes problematic use of digital tools.

The concept of Problematic Internet Use (PIU) was developed to describe situations in which the use of digital tools goes beyond instrumental use and leads to negative effects on psychological and social well-being (Young, 1998; Davis, 2001; Caplan, 2002). In this context, the instrument developed by Young (1998), the Internet Addiction Test (IAT), is still one of the most frequently used indicators for measuring PIU including in the educational sector. This phenomenon is particularly relevant for adolescents, as they are more susceptible to forms of addiction and excessive technology use due to their stage of development (Waldo, 2014). According to Davis (2001), users with a tendency towards PIU are more likely to approach and quickly familiarize themselves with innovative digital tools, which reinforces constant usage habits. This link makes PIU a relevant construct to integrate into models such as UTAUT2, as it can reinforce dimensions such as habit formation for regular and routine use of AI (Kuss and Griffiths, 2011).

A second important aspect concerns attitudes towards the use of technology and, in the case of this study, towards AI (Attitude Toward Artificial Intelligence, ATAI). Recent research has developed instruments that specifically measure these attitudes and show how they can influence the intention to use AI (Sindermann et al., 2021). However, as Turós et al. (2025) has shown in a study of high school students, positive attitudes towards AI do not necessarily translate into actual repeated or routine use, especially when it comes to school activities such as completing assignments. This discrepancy suggests that ATAI, when integrated into the UTAUT2 framework, can contribute to a better understanding of the differences between intention, actual use and habit formation.

The integration of PIU and ATAI within the UTAUT2 framework in the present study therefore aims to provide a more comprehensive perspective on the psychological and motivational factors that influence the development of habitual AI use and future adoption intentions in high school students. See Table 1 for a list of the constructs included in the model, and their definition.

**Table 1:** Key constructs of the extended UTAUT2 framework including PIU and ATAI.

Construct	Description	Source
Performance expectancy	The degree of the belief that using the technology improves performance.	Venkatesh et al. (2003)
Effort expectancy	The degree of ease associated with the use of the system.	Venkatesh et al. (2003)
Social influence	The degree to which an individual perceives that important others believe they should use the system.	Venkatesh et al. (2003)
Facilitating conditions	The degree to which an individual believes that organizational and technical infrastructure exists to support use of the system.	Venkatesh et al. (2003)
Hedonic motivation	The fun or pleasure derived from using the technology.	Venkatesh et al. (2012)
Price value	The trade-off between the perceived benefits and the monetary or data privacy costs of using the technology.	Venkatesh et al. (2012)
Habit	The extent to which people tend to perform behaviors automatically because of learning.	Venkatesh et al. (2012)
Behavioural intention	The degree to which an individual has formulated conscious plans to perform or continue using the behavior in the future.	Venkatesh et al. (2003)
Problematic internet use (PIU)	The tendency toward compulsive and excessive Internet use, which can reinforce habitual patterns of technology use.	Young (1998), Davis (2001), and Caplan (2002)
Attitude toward AI (ATAI)	The general attitude and evaluation of artificial intelligence as a supportive tool.	Sindermann et al. (2021)

### The present study

Although the UTAUT2 provides an effective framework for the systematic investigation of the factors influencing technology adoption – while offering the possibility to include contextual and demographic factors such as gender, school type or cultural background to reflect the specific educational environment (Tamilmani et al., 2021; Soliman et al., 2025) – its application remains limited to the study of AI use among high school students (Strzelecki, 2024).

A second aspect that is still insufficiently developed concerns the integration of psychological constructs within this framework. While some previous studies have considered dimensions such as technology-related anxiety (Li et al., 2024; Chai et al., 2020), the integration of PIU (Young, 1998; Davis, 2001; Caplan, 2002) and ATAI (Sindermann et al., 2021) into the UTAUT2 in the context of AI adoption among adolescents is still poorly explored (Huang et al., 2024; Turós et al., 2025). Further research is therefore needed to understand how PIU can act as an indicator of compulsive behavior and risks to digital wellbeing, and how ATAI translates into sustained Behavioural adoption.

In light of the previous considerations, this study extends the UTAUT2 framework to investigate AI use among Italian high school students, examining how demographic characteristics, school contexts, and psychological variables influence two key outcomes: habitual use of AI technologies and the intention to use them. Specifically, we investigate the role of PIU and ATAI, providing a more comprehensive model of technology adoption. PIU captures behavioural compulsivity, reflecting usage patterns rooted in habit and dependence, while ATAI addresses the motivational and evaluative aspects that influence intentional engagement. Together, they provide a nuanced understanding of how external (e.g., social pressure, facilitating conditions) and internal (e.g., attitudes, compulsive tendencies) factors interact to shape AI adoption behaviors.

In addition, the study examines high school students’ current AI use patterns as a significant factor driving habit formation and behavioural intention to use AI in the future. High school students’ current AI use reflects how these technologies are already being integrated into their daily lives, providing a foundation for habitual engagement and future use. Whether AI is used for practical, creative, or academic purposes, frequent interaction in these contexts is likely to reinforce habits through positive reinforcement and shape their willingness to continue or expand their usage in the future.

Specifically, this research addresses the following objectives:

1. To assess the applicability of the UTAUT2 framework to high school student populations in the context of AI technologies, evaluating the relevance of constructs like Performance Expectancy, Effort Expectancy, Hedonic Motivation, and Facilitating Conditions, while incorporating psychological and contextual moderators specific to this demographic.
2. To identify the factors influencing habitual use and behavioural intentions related to AI technologies among Italian high school students, including individual demographic traits, school-related contextual variables, and psychological constructs such as Problematic Internet Use (PIU) and Attitudes Toward AI (ATAI) and current AI use.

In exploring these aims, the present study adopts a non-experimental, correlational approach leveraging questionnaire data. We aim to provide novel findings by extending the UTAUT2 framework through an innovative examination of AI technology adoption among high school students. By integrating demographic variables and psychological constructs within a validated technological acceptance model, the present study aims to offer a more comprehensive understanding of how student’s individual characteristics collectively influence AI usage habits and behavioural intentions.

## Method

### Participants

The initial sample comprised 1,035 high school students aged between 14 and 20 years (M = 16.18, SD = 1.28); 55.88% identified as female, 1.40% as non-binary, and 85.50% reported Italian citizenship. A total of 102 participants completed only the demographic section and were therefore excluded from subsequent analyses. The resulting study sample consisted of 933 high school students from Italy, aged between 14 and 20 years old (*M* = 16.20, *SD* = 1.29). Among the participants, 54.98% identified as female, 1.29% identified as non-binary, and 85.64% indicated they were Italian citizens. The largest proportion of students (51.8%) attends a lyceum, students from vocational schools make up 26.8%, while those in technical schools form the smallest group at 21.4%.

Participants completed the questionnaire in their classrooms; to address potential biases inherent in self-report data, we assured participants of anonymity and confidentiality and clearly stated there were no right or wrong answers. This approach aimed to reduce social desirability and encourage honest responses. Participation in this study was completely voluntary and carried no incentives or rewards. Note that all instruments were pre-tested with a pilot group of 25 students to ensure clarity and age-appropriateness of the adapted items. Based on student feedback, no adjustments were required to the instrument, as it was deemed comprehensible, relevant, and suitable for the target age group. Prior to the data collection, ethical approval was obtained from the ethics committee at the authors’ affiliated university (IRB N. 856,434).

### Measures

#### Unified theory of acceptance and use of technology 2

To assess the determinants of AI use, this study employed an adapted version of the Unified Theory of Acceptance and Use of Technology 2 (UTAUT2) model (Venkatesh et al., 2012). UTAUT2 is a robust framework for understanding technology acceptance, encompassing key constructs such as Performance Expectancy, Effort Expectancy, Social Influence, Facilitating Conditions, Hedonic Motivation, Price Value, Habit, and Behavioural Intention. The original UTAUT2 items were modified to specifically address the context of AI use in this study. For instance, phrases like “mobile Internet” in the original questionnaire were replaced with “Artificial Intelligence.” Participants responded to each item using a 5-point Likert scale ranging from 1 (Strongly Disagree) to 5 (Strongly Agree). Example items include: “I believe that artificial intelligence is useful in my studies” (Performance Expectancy); “Learning to use artificial intelligence is easy for me” (Effort Expectancy); “People who influence my behavior believe I should use artificial intelligence” (Social Influence); and “Using artificial intelligence is fun” (Hedonic Motivation).

#### AI usage patterns

First, we asked participants to report on their currently used AI tools using an open-ended question [“Which artificial intelligence applications do you use (e.g., ChatGPT, Google Gemini, Algor Education, etc.)?”]. Based on manual coding of student responses, ChatGPT was the most widely used tool, with 68.6% of respondents reporting its use. This was followed by Google Translate at 4.7%, Bing Copilot at 3.7%, Google Gemini at 3.2%, Photomath at 3.0%, and MyAI Snapchat at 2.9%. Reverso and Canva both had a usage rate of 1.4%, while Algor and Character each had 0.8%. PizzaGPT was used by 0.5% of participants, and Claude had the lowest usage rate, at just 0.1%.

Next, we asked students to report on of how they you used artificial intelligence (e.g., for translation, creating texts, doing school assignments, creating images for social media, etc.). The analysis of responses revealed that 53.59% of students reported using AI for school support or homework tasks, 38.59% for content creation, 36.98% for translation, and 22.72% for information search or research. These variables were measured as binary indicators (0 = no, 1 = yes), capturing whether participants engaged in each specific AI use category.

#### Problematic internet use

The study employed an adaptation of the 8-item instrument designed by Young (1998) to assess participants’ tendencies toward Problematic Internet Use (PIU). This instrument includes items designed to explore behaviors and attitudes related to Internet use. Example items are: “Do you feel the need to use the Internet with increasing amounts of time in order to achieve satisfaction?”; [“Do you use the Internet as a way of escaping from problems or of relieving a dysphoric mood (e.g., feelings of helplessness, guilt, anxiety, depression)?”]. Participants were asked to reflect on the frequency of the behaviors described in each question and select the response that best described their situation, using the following scale: 0 = Never, 1 = Rarely, 2 = Occasionally, 3 = Often, 4 = Very Often. For the present study, Cronbach’s Alpha was acceptable (*α* = 0.78).

#### Attitude towards artificial intelligence

To assess participants’ attitudes towards artificial intelligence, we adapted the Attitude Towards Artificial Intelligence (ATAI) scale developed by Sindermann et al. (2021). This scale is designed to measure general attitudes towards AI across different cultural contexts. The ATAI scale consists of five items presented as statements related to AI, with which participants indicate their level of agreement on a 5-point Likert scale ranging from 1 (strongly disagree) to 5 (strongly agree). The items capture a range of sentiments towards AI, including fear, trust, perceived existential threat, potential benefits, and concerns about job displacement: 1. AI frightens me; 2. I trust AI; 3. AI will destroy humanity.; 4. AI will benefit humanity; 5. AI will cause the loss of many jobs. The scale’s brevity and straightforward wording make it suitable for use with diverse populations, including high school students. For the present study, Cronbach’s Alpha was acceptable (α = 0.70).

### Data analysis

To assess the applicability of the UTAUT2 model to AI use, Confirmatory Factor Analysis (CFA) was performed. This analysis evaluated the structural validity of the model, focusing on factor loadings, covariances, and standard model fit indices, including Chi-square (χ^2^), Comparative Fit Index (CFI), Tucker-Lewis Index (TLI), Standardized Root Mean Square Residual (SRMR), and Root Mean Square Error of Approximation (RMSEA). We consider values of CFI > 0.95, TLI > 0.95, and RMSEA < 0.05 as an indication of good model fit, while CFI and TLI > 0.90, and RMSEA < 0.08, as indication of acceptable fit (Hu and Bentler, 1999; Marsh et al., 2004).

Reliability was assessed using Cronbach’s alpha (α) and McDonald’s Omega (*ω*) to ensure internal consistency of the constructs. These analyses were conducted in R using the *lavaan* (Rosseel, 2012) and *psych* (Revelle, 2019) packages.

Subsequently, multiple regression analyses were conducted to investigate the predictors of Habit and Behavioural Intention related to AI use as outcome variables. The predictors included demographic characteristics (e.g., age, gender, Italian citizenship, type of secondary school), student’s GPA, type of AI usage contexts (e.g., translation, information retrieval, schoolwork, content creation), PIU, ATAI, and the key constructs from the UTAUT2 model (i.e., Performance Expectancy, Effort Expectancy, Social Influence, Facilitating Conditions, Hedonic Motivation, Price Value). The regression models were designed to assess the unique contribution of each predictor while statistically controlling for potential confounding variables. Standardized beta coefficients (*β*) were used to interpret effect sizes, with model fit evaluated using R^2^ and Adjusted R^2^ to account for explained variance and model complexity. Significance was determined at *p* < 0.05. These analyses were performed in SPSS, version 29.

## Results

### Model fit of UTAUT2

The chi-square test was significant (χ^2^ = 1,152, df = 322, *p* < 0.001), which is expected with large samples. However, other fit indices indicated acceptable model fit: CFI = 0.94, TLI = 0.93, SRMR = 0.0507, RMSEA = 0.0516 (90% CI [0.0484, 0.0549]). All items had standardized loadings >0.50, except for Item 15 (“I can get help from others when I have difficulties using artificial intelligence”) from the Facilitating Conditions dimension that had a factor loading = 0.20. We decided to remove the item. After running the model a second time, the chi-square test was still significant (χ^2^ = 1,050, df = 296, *p* < 0.001). Fit indices indicated acceptable model fit: CFI = 0.95, TLI = 0.94, SRMR = 0.0485, RMSEA = 0.0513 (90% CI [0.048, 0.0547]).

Table 2 presents the factor loadings for the retained items. All standardized factor loadings were above 0.50, indicating strong relationships between the items and their respective constructs. All loadings were statistically significant (*p* < 0.001). All factor correlations were statistically significant (*p* < 0.001), indicating significant relationships between the constructs, with latent correlations ranging from 0.246 to 0.801.

**Table 2:** Standardized factor loadings for the UTAUT 2 dimensions.

Factor	Indicator	Stand. estimate
Performance expectancy	I find Artificial Intelligence useful in my studies	0.801
	Using Artificial Intelligence increases my chances of achieving things in my studies	0.801
	Using Artificial Intelligence helps me accomplish things more quickly in my studies	0.659
	Using Artificial Intelligence increases my productivity in my studies	0.723
Effort expectancy	Learning how to use Artificial Intelligence is easy for me	0.830
	My interaction with Artificial Intelligence is clear and understandable	0.718
	I find Artificial Intelligence easy to use	0.793
	It is easy for me to become skillful at using Artificial Intelligence	0.816
Social influence	People who are important to me think that I should use Artificial Intelligence	0.807
	People who influence my behavior think that I should use Artificial Intelligence	0.882
	People whose opinions that I value prefer that I use Artificial Intelligence	0.883
Facilitating conditions	I have the resources necessary to use Artificial Intelligence	0.755
	I have the knowledge necessary to use Artificial Intelligence	0.798
	Artificial Intelligence is compatible with other technologies I use	0.559
Hedonic motivation	Using Artificial Intelligence is fun	0.868
	Using Artificial Intelligence is enjoyable	0.863
	Using Artificial Intelligence is very entertaining	0.811
Price value	Artificial Intelligence is reasonably priced	0.769
	Artificial Intelligence is a good value for the money	0.875
	At the current price, Artificial Intelligence provide a good value	0.769
Habit	The use of Artificial Intelligence has become a habit for me	0.851
	I am addicted to using Artificial Intelligence services	0.748
	I must use Artificial Intelligence	0.738
	Using Artificial Intelligence has become natural to me	0.860
Behavioural intention	I intend to continue using Artificial Intelligence in the future	0.758
	I will always try to use Artificial Intelligence in my school studies	0.797
	I plan to continue to use Artificial Intelligence frequently	0.873

Cronbach’s alpha coefficients were calculated to assess the reliability of the constructs, with all values meeting acceptable thresholds. Performance Expectancy showed an alpha of 0.829 (McDonald’s *ω* = 0.832), and Effort Expectancy had 0.867 (McDonald’s ω = 0.869). Social Influence and Hedonic Motivation exhibited strong reliability with alphas of 0.892 (McDonald’s ω = 0.893) and 0.886 (McDonald’s ω = 0.888), respectively. Price value and Habit also showed high reliability, with alphas of 0.844 (McDonald’s ω = 0.847) and 0.879 (McDonald’s ω = 0.888). Behavioural Intention had an alpha of 0.846 (McDonald’s ω = 0.849), while Facilitating Conditions reported a slightly lower but acceptable alpha of 0.737 (McDonald’s ω = 0.750). These results indicate that the constructs demonstrate consistent internal reliability.

### Regression analyses predicting habit and behavioural intention related to AI use

The regression analyses revealed distinct predictors for habit and behavioural intention, with model fit indices suggesting varying explanatory power (see Table 3). The model for Habit explained 36% of the variance (R^2^ = 0.36; Adj. R^2^ = 0.35), while the model for behavioural intention explained 50% (R^2^ = 0.50; Adj. R^2^ = 0.49).

**Table 3:** RegressIon analyses predicting UTAUT 2 habit and behavioural intention related to AI use.

Predictor	Habit (R^2^ = 0.36; Adj R^2^ = 0.35)		Behavioural intention (R^2^ = 0.50; Adj R^2^ = 0.49)	
	β	*p*	β	*p*
Age	−0.044	0.112	0.016	0.506
Non-binary (gender)	−0.018	0.517	0.012	0.622
Female (gender)	0.053	0.089	0.011	0.684
Italian (citizen)	−0.075	0.008	−0.001	0.979
Type of school: lyceum	−0.173	< 0.001	−0.042	0.162
Type of school: technical school	−0.069	0.035	0.001	0.986
GPA	−0.039	0.185	−0.043	0.091
AI use: translation	0.034	0.228	−0.004	0.870
AI use: information retrieval	0.027	0.324	0.046	0.054
AI use: Schoolwork-related	0.075	0.010	0.136	< 0.001
AI use: content creations	−0.042	0.131	0.034	0.166
UTAUT2: performance expectancy	0.165	< 0.001	0.362	< 0.001
UTAUT2: effort expectancy	0.058	0.128	−0.011	0.746
UTAUT2: social influence	0.262	< 0.001	0.151	< 0.001
UTAUT2: facilitating conditions	0.004	0.922	0.092	0.005
UTAUT2: hedonic motivation	0.104	0.002	0.126	< 0.001
UTAUT2: price value	0.048	0.105	0.039	0.139
Attitude towards AI	−0.013	0.666	0.085	0.002
Problematic Internet Use	0.172	< 0.001	0.097	< 0.001

β: standardized regression coefficients are reported.

For Habit, significant positive predictors included performance expectancy (*β* = 0.165, *p* < 0.001), social influence (β = 0.262, *p* < 0.001), Hedonic Motivation (β = 0.104, *p* = 0.003), Schoolwork-related use of AI (β = 0.075, *p* = 0.010), and Problematic Internet Use (PIU) scores (β = 0.172, *p* < 0.001). Students reporting having an Italian citizenship showed lower Habit compared to students with non-Italian background (β = −0.075, *p* = 0.008). Additionally, when compared with students attending vocational school, student attending a lyceum (β = −0.173, *p* < 0.001), or a Technical school (β = −0.069, *p* = 0.038) report lower habit related to AI use. Interestingly, Attitude Towards AI (ATAI) was not a significant predictor for Habit (β = −0.013, *p* = 0.666).

For behavioural intention, significant positive predictors were performance expectancy (β = 0.362, *p* < 0.001), social influence (β = 0.151, *p* < 0.001), hedonic motivation (β = 0.126, *p* < 0.001), facilitating conditions (β = 0.092, *p* = 0.005), schoolwork-related use of AI (β = 0.136, *p* < 0.001), PIU (β = 0.097, *p* < 0.001), and ATAI (β = 0.085, *p* = 0.002). While information retrieval approached significance (β = 0.046, *p* = 0.057), other predictors such as effort expectancy, price value, and demographic variables did not significantly contribute to the model.

These results suggest that habitual AI use is primarily driven by social and performance-based factors, as well as enjoyment and task relevance. However, behavioural intention appears to be more broadly influenced, with ATAI emerging as a significant predictor, highlighting its importance in shaping user intentions. The continued influence of facilitating conditions and social factors underscores the role of external support and community in encouraging AI adoption.

## Discussion

The present study provides a comprehensive examination of adoption of artificial intelligence (AI) technology among Italian high school students, extending the Unified Theory of Acceptance and Use of Technology (UTAUT2) framework through an innovative analysis of psychological and contextual factors influencing AI usage habits and behavioural intentions (Tamilmani et al., 2021).

Our research revealed nuanced insights into AI technology adoption among adolescents. The model for habit and behavioural intention demonstrated significant explanatory power, highlighting the complex interplay of factors shaping AI technology engagement. Notably, Performance Expectancy consistently showed strong positive associations with both habit and behavioural intention for AI use, suggesting a significant association between students’ perception of the benefits of AI and their level of technological engagement. The substantial effect on behavioural intention indicates that perceived instrumental value may play a crucial role in shaping future technology use intentions (Grassini et al., 2024; Moravec et al., 2024).

Social Influence also emerged as a key variable for both habit and behavioural intention, underscoring the profound impact of peer dynamics and social contexts on technological adoption (Ryzhko et al., 2023). This finding aligns with previous research emphasizing the role of social networks in technology acceptance, particularly among younger populations (Ali et al., 2024). A perceived influence resulting from the behavior of peers seems to play an important role: when students observe or sense that their classmates are actively using AI, they may feel encouraged or indirectly motivated to adopt similar practices. This tendency may be reinforced by a fear of falling behind, whether through a desire to acquire technological skills that improve academic performance and provide a competitive advantage, or by conforming to social norms, trends and habits that are seen as essential for social integration and success.

Findings on Hedonic Motivation suggest that it has significant, though comparatively weaker, associations with both habit and behavioural intention to use AI. This indicates that while most students reported using AI primarily for academic purposes, such as homework support, the perceived value of AI use may also be influenced by the intrinsic enjoyment it provides (Shuhaiber et al., 2025). The remaining UTAUT2 variables showed mixed or non-significant effects on habit and behavioural intention related to AI use. Among these, Facilitating Conditions showed a significant association with behavioural intention but not of habit formation. This finding aligns with previous research highlighting the importance of initial support mechanisms in fostering users’ intention to adopt a technology (Habibi et al., 2023). In contrast, other components of the model—namely social influence, performance expectancy, and hedonic motivation—appear to serve as stronger psychological drivers of habit formation.

Effort Expectancy was not associated with behavioural intention, possibly reflecting the intuitive and user-friendly nature of AI tools such as ChatGPT, which require minimal learning effort. This may be particularly relevant for adolescents, often referred to as “digital natives” (Prensky, 2001), who have grown up immersed in digital environments. Their continuous exposure to technology has likely shaped distinct cognitive patterns and usage habits, making it easier for them to adopt new tools that demand little additional effort. Price value also did not reveal significant associations with either habit or behavioural intention, an outcome that warrants further consideration. According to Venkatesh et al. (2012) and supported by the meta-analysis of Tamilmani et al. (2021), price value is typically conceptualized in terms of direct monetary cost, reflecting users’ evaluation of whether the benefits of a technology justify its financial expense. Some students may have interpreted this construct narrowly, considering only the economic cost and deeming it irrelevant due to their use of free or freemium AI tools like ChatGPT. Others may have adopted a broader interpretation, viewing “cost” in terms of data sharing or providing feedback in exchange for usage (LaRose et al., 2008; Soliman et al., 2025). This divergence in interpretation may partly account for the non-significant association between price value and habit and behavioural intention related to AI use.

Looking beyond the examined UTAUT2 variables, we found several significant associations with habit and behavioural intention to use AI. Among these, schoolwork-related AI use was positively associated with both habit formation and future behavioural intention related to AI use, suggesting that educational utility is a primary mechanism for use of AI technology, which in turn it’s consistent with the observed surge in school-related use of LLMs among students secondary education (Zhu et al., 2025). In contrast, use of AI for content creation and translation showed non-significant associations in relation to habit and behavioural intention to use AI, though both represented relevant usage categories in our sample. This suggests that while students actively employ AI for these purposes, such applications may not be as strongly linked to long-term technological engagement.

The study also revealed interesting contextual variations. Students with non-Italian backgrounds showed higher AI usage habits, suggesting cultural and potentially socioeconomic factors influence technological adoption. This aligns with findings that international students, who often face cultural and language barriers, may be more inclined to use AI tools to support their academic performance (Ittefaq et al., 2025). Beyond this, students from vocational also demonstrated higher habit tendencies related to AI compared to those in technical and lyceum schools, indicating potential institutional variations in technological engagement (Baek et al., 2024). These findings suggest that AI use is unlikely to be uniform, but rather contextual, and may be influenced by the goals, expectations, and challenges specific to each student group (Acosta-Enriquez et al., 2024).

Of note, students’ attitude towards AI (ATAI) showed a positive association with behavioural intention but not Habit formation, suggesting that general attitudes toward AI support initial adoption decisions but are not sufficient to sustain habitual use (Sindermann et al., 2021; Turós et al., 2025). This reinforces prior findings showing that attitudes influence intention but must be supported by reinforcement mechanisms to shape long-term behavior. Finally, an intriguing and novel finding from the present study indicated that Problematic Internet Use (PIU) significantly was significantly associated with both habit and behavioural intention related to AI use. This finding aligns with the existing literature indicating that PIU is strongly associated with a higher propensity to adopt and engage with new technologies, extending this concept into the domain of artificial intelligence. Prior research has identified PIU as a predictor of early adoption and increased use of digital platforms, often motivated by novelty seeking, escapism, and social compensation (Kuss and Griffiths, 2017; Montag et al., 2021). The current study builds on this foundation by demonstrating that these same psychological drivers may now be directed towards AI, resulting in habitual engagement with AI tools and intentional efforts to integrate AI into everyday life. This extends our understanding of PIU by suggesting a spillover effect, where problematic online behaviors do not remain confined to traditional Internet usage but adapt to incorporate use of emerging technologies like AI, not necessarily for functional outcomes but as a coping mechanism (Caplan, 2002; Davis, 2001). In turn, excessive AI use, possibly fueled by PIU, could hinder the development of critical cognitive skills such as planning, problem-solving, and independent thinking (Lai et al., 2023; Xie et al., 2022). This represents a paradox: AI is used to improve performance, but its overuse may diminish the very capacities needed for autonomous learning. These interpretations must be treated with caution due to the cross-sectional design. It remains unclear whether compulsive engagement with AI stems from general PIU or from specific psychological effects unique to human-like AI systems (Sindermann et al., 2021; Turós et al., 2025).

From a theoretical standpoint, this study confirms the validity of UTAUT2 for modeling adolescent AI adoption, consistent with findings in university populations (Soliman et al., 2024; Tamilmani et al., 2021). The results also demonstrate the added value of integrating psychological constructs into the model, especially PIU, which helps explain compulsive behavior patterns not captured by UTAUT2 alone. Furthermore, this study directly addressed the research gaps described in the literature review. While UTAUT2 has been widely used to explain the adoption of technology in general (Tamilmani et al., 2021), its application to the use of AI among high school students is still limited (Strzelecki, 2024). By testing UTAUT2 in this specific context, the study provides new evidence that key constructs such as Performance Expectancy and Social Influence are strongly associated with behavioural intention and habit, supporting the model’s original assumptions and extending its relevance to high school students population. In addition, the study also fills the gap created by the limited integration of psychological constructs into the UTAUT2 framework for high school students, capturing the role of compulsive tendencies and attitudes that the standard UTAUT2 model does not fully explain (Huang et al., 2024).

In view of the observed associations between perceived utility of AI, social influence and the potential for problematic use patterns, promoting balanced and conscious use of AI among high school students appears to require carefully designed interventions. International guidelines such as the UNESCO AI Competency Frameworks for teachers and students (Miao and Cukurova, 2024) provide valuable guidance for the development of AI competency initiatives that go beyond technical education and promote a critical, ethical and human-centered understanding of AI systems. These frameworks emphasize the importance of students becoming not only informed users, but also responsible co-creators who are aware of both the opportunities and risks associated with the use of AI. At the same time, the role of teachers as learning guides is fundamental and could be strengthened accordingly. As recent pedagogical approaches such as Situated Learning Episodes with AI (ESLAI) (Panciroli et al., 2023) and the Artificial Intelligence-Technological Pedagogical Content Knowledge (AI-TPACK, Ning et al., 2024) show, teacher education may benefit from integrating AI into a robust instructional design framework that combines disciplinary knowledge, pedagogical strategies and computational thinking. The Synergy between People and Artificial Intelligence for Collaborative Education (S.P.Ai.C.E.) model (Messina and Panciroli, 2025) supports this approach by providing educators with tools to critically select, test and validate AI applications in rapidly evolving technological contexts. From a practical perspective, the deliberate and collaborative use of AI can support teachers in implementing established methodological approaches by acting as a didactic mediator. For example, within the consolidated framework of Universal Design for Learning (CAST, 2018), AI might be used to create accessible teaching materials such as concept maps (Novak and Gowin, 1984) and mind maps (Buzan and Buzan, 1993), as well as to produce text simplifications or translate learning content for students who are not yet fully proficient in the language of instruction. Such concrete applications may allow teachers to tailor the content to the different needs of the students while maintaining the pedagogical depth of the lesson.

## Limitations and future research

While our study provides valuable insights, several limitations warrant acknowledgment. First, the study employed a cross-sectional design, which limits the ability to draw causal inferences regarding the observed relationships among psychological, contextual, and technological variables. Although our model identifies significant predictors of habitual and intentional AI use, longitudinal studies are needed to establish the temporal ordering and directionality of these associations.

Second, the exclusive reliance on self-report measures introduces the potential for common-method bias. Despite efforts to minimize social desirability effects, such as guaranteeing anonymity and emphasizing the absence of right or wrong answers, the use of a single method of data collection may have inflated some of the associations among variables. Future research would benefit from employing multi-method approaches, including behavioural data (e.g., logs of AI usage), peer or teacher reports, or experimental paradigms, to strengthen the validity of findings and reduce method variance.

Lastly, given the rapid evolution of AI technologies and their applications in education, future studies should adopt dynamic and iterative research designs to capture changes in usage patterns and psychological correlates over time. Incorporating qualitative methodologies may also help uncover deeper motivations and concerns that underlie adolescent engagement with AI, providing richer context for interpreting quantitative findings.

## Conclusion

By integrating psychological constructs within the UTAUT2 framework, this research advances the understanding of AI technology adoption among adolescents. The findings emphasize the multifaceted nature of technological engagement, highlighting the interplay of social, psychological, and contextual factors in shaping digital behaviors.

The study confirms the central role of Social Influence, Performance Expectancy, and Hedonic Motivation in driving both behavioural intention and habit, underscoring how peer networks and the perceived utility and pleasantness of AI significantly impact technology adoption. Notably, the use of AI as a tool for enhancing academic performance, such as completing school assignments, reinforces the importance of guiding students toward a critical and mindful engagement with these technologies.

A significant and novel contribution of this study lies in the identification of the spillover effect from PIU to AI adoption. This relationship highlights how dependency-driven behaviors may influence habitual AI engagement, shifting its use from a functional academic tool to a form of digital escapism. While AI’s utilitarian potential seems to align with students’ performance expectations and may support the achievement of academic goals, the shadow of compulsive and problematic use could pose a critical risk that should not be overlooked. Such risks might include negative consequences for students’ wellbeing, learning processes, and overall cognitive development. This finding underscores the need for targeted strategies and policies to foster a balanced relationship with AI, ensuring that its adoption supports learning and academic success without compromising students’ cognitive development or digital wellbeing. Educational institutions and teachers are at the forefront of this effort. They should aim to equip students with the skills to critically evaluate AI-generated content, understand its limitations – including biases and inaccuracies – and maintain human oversight. Most of all, they should focus on helping students develop an awareness of the potential risks associated with problematic AI use, which could negatively impact their learning, health, and wellbeing. Integrating AI into targeted educational activities could help clarify its role as a tool that complements, rather than replaces, students’ learning processes.

Looking ahead, this research opens several avenues for future studies. Cross-cultural comparisons, subgroups analysis, longitudinal analyses, and objective measures of AI usage would provide a deeper understanding of how technological habits evolve over time. Moreover, exploring the perspectives of teachers and the adoption of AI for pedagogical purposes could yield practical insights for designing effective interventions and training programs.

## Data Availability

The raw data supporting the conclusions of this article will be made available by the authors, without undue reservation.
