# Supplementary material for: AI adoption among adolescents in education: extending the UTAUT2 with psychological and contextual factors
Source: Front Artif Intell. 2025 Sep 8;8:1614993. doi: 10.3389/frai.2025.1614993 (PMC12451006; doi:10.3389/frai.2025.1614993)
Supplement: Supplementary file 1 [file Table_1.docx]

**Supplementary material**

**Table S1**. Regression Analyses predicting UTAUT 2 Habit related to AI use (R^2^ = .36; Adj R^2^ = .35)

|  |  |  |  | 95% CI | |  |
| --- | --- | --- | --- | --- | --- | --- |
|  | b | SE | p | LL | UL | β |
| Age | -.032 | .020 | .112 | -.071 | .007 | -.044 |
| Non-Binary(Gender) | -.149 | .230 | .517 | -.600 | .302 | -.018 |
| Female (Gender) | .102 | .060 | .089 | -.016 | .220 | .053 |
| Italian (Citizen) | -.204 | .076 | .008 | -.354 | -.054 | -.075 |
| Type of School: Lyceum | -.330 | .065 | < .001 | -.456 | -.203 | -.173 |
| Type of School: Technical school | -.159 | .075 | .035 | -.307 | -.011 | -.069 |
| GPA | -.042 | .032 | .185 | -.104 | .020 | -.039 |
| AI Use: Translation | .067 | .056 | .228 | -.042 | .176 | .034 |
| AI Use: Information retrieval | .062 | .062 | .324 | -.061 | .184 | .027 |
| AI Use: Schoolwork-related | .144 | .056 | .010 | .035 | .254 | .075 |
| AI Use: Content Creations | -.083 | .055 | .131 | -.190 | .025 | -.042 |
| UTAUT2: Performance Expectancy | .191 | .042 | < .001 | .109 | .273 | .165 |
| UTAUT2: Effort Expectancy | .071 | .047 | .128 | -.020 | .162 | .058 |
| UTAUT2: Social Influence | .282 | .033 | < .001 | .216 | .347 | .262 |
| UTAUT2: Facilitating Conditions | .005 | .050 | .922 | -.093 | .102 | .004 |
| UTAUT2: Hedonic Motivation | .121 | .039 | .002 | .044 | .198 | .104 |
| UTAUT2: Price Value | .060 | .037 | .105 | -.012 | .132 | .048 |
| Attitude towards AI | -.020 | .046 | .666 | -.111 | .071 | -.013 |
| Problematic Internet Use | .241 | .040 | < .001 | .163 | .320 | .172 |
| Note. CI: Confidence interval; LL: Lower limit; UL: Upper limit. | | | | | | |

**Table S2**. Regression Analyses predicting UTAUT 2 Behavioural Intention related to AI use (R2 = .50; Adj R2 = .49)

|  |  |  |  | 95% CI | |  |
| --- | --- | --- | --- | --- | --- | --- |
|  | b | SE | p | LL | UL | β |
| Age | .012 | .018 | .506 | -.023 | .047 | .016 |
| Non-Binary(Gender) | .102 | .206 | .622 | -.302 | .505 | .012 |
| Female (Gender) | .022 | .054 | .684 | -.084 | .127 | .011 |
| Italian (Citizen) | -.002 | .068 | .979 | -.136 | .132 | -.001 |
| Type of School: Lyceum | -.081 | .058 | .162 | -.194 | .033 | -.042 |
| Type of School: Technical school | .001 | .067 | .986 | -.131 | .133 | .001 |
| GPA | -.048 | .028 | .091 | -.104 | .008 | -.043 |
| AI Use: Translation | -.008 | .050 | .870 | -.106 | .089 | -.004 |
| AI Use: Information retrieval | .107 | .056 | .054 | -.002 | .217 | .046 |
| AI Use: Schoolwork-related | .263 | .050 | < .001 | .166 | .361 | .136 |
| AI Use: Content Creations | .068 | .049 | .166 | -.028 | .164 | .034 |
| UTAUT2: Performance Expectancy | .425 | .038 | < .001 | .351 | .498 | .362 |
| UTAUT2: Effort Expectancy | -.013 | .042 | .746 | -.095 | .068 | -.011 |
| UTAUT2: Social Influence | .165 | .030 | < .001 | .106 | .223 | .151 |
| UTAUT2: Facilitating Conditions | .126 | .044 | .005 | .039 | .213 | .092 |
| UTAUT2: Hedonic Motivation | .148 | .035 | < .001 | .079 | .216 | .126 |
| UTAUT2: Price Value | .049 | .033 | .139 | -.016 | .113 | .039 |
| Attitude towards AI | .132 | .041 | .002 | .051 | .214 | .085 |
| Problematic Internet Use | .137 | .036 | < .001 | .067 | .207 | .097 |
| Note. CI: Confidence interval; LL: Lower limit; UL: Upper limit. | | | | | | |
